# Comparison of Clinical Efficacy Between Interlaminar and Transforaminal Epidural Injection in Patients With Axial Pain due to Cervical Disc Herniation

**DOI:** 10.1097/MD.0000000000002568

**Published:** 2016-01-29

**Authors:** Jung Hwan Lee, Sang-Ho Lee

**Affiliations:** From the Departments of Physical Medicine and Rehabilitation (JHL) and Neurosurgery (S-HL), Wooridul Spine Hospital, Seoul, Korea.

## Abstract

Transforaminal (TF) approach is preferred by physician to interlaminar (IL) approach because it can deliver injectates directly around nerve root and dorsal root ganglion, which is regarded as main pain sources. Axial neck pain is originated from sinuvertebral nerve located in ventral epidural spaces, which has been described to be related to central or paramedian disc herniation. It is very questionable that TF injection is also more effective than IL injection in the patients with axial neck or interscapular pain. This study was to evaluate clinical efficacy of cervical epidural injection in patients with axial pain due to cervical disc herniation and to compare the clinical outcomes between TF and IL approaches. Fifty-six and 52 patients who underwent IL and TF epidural injections, respectively, for axial neck/interscapular pain due to central or paramedian cervical disc herniation were included. Numeric Rating Scale (NRS) and Neck Disability Index (NDI) were compared between both groups at 2 and 8 weeks after treatment. Successful pain relief was defined if a 50% or more reduction of NRS score was achieved in comparison with pretreatment one. Successful functional improvement was defined if at least a 40% reduction of NDI was obtained. Overall, 79 (73.1%) and 57 (52.8%) among 108 patients showed successful pain relief at 2 and 8 weeks, respectively. Seventy-six (70.4%) and 52 (48.1%) had successful functional improvement at 2 and 8 weeks, respectively. The IL and TF groups showed no significant difference in proportion of successful results of NRS 2 weeks (73.2% vs 67.3%) and 8 weeks (48.2% vs 48.1%). Also, no significant difference was obtained in proportion of successful NDI between 2 groups at 2 weeks (75.0% vs 71.2%) and 8 weeks (53.6% vs 51.9%). Cervical epidural injection showed favorable results in 2 weeks and moderate results in 8 weeks in patients with axial pain due to cervical disc herniation. IL and TF showed no significant difference in clinical efficacy. Considering TF was relevant to more serious side effects, IL was more recommendable in these patients.

## INTRODUCTION

Cervical epidural steroid injection using transforaminal (TF) or interlaminar (IL) approach has been conducted to control neck or radicular pain, which is caused by cervical herniated disc or stenosis. There have been several literatures about clinical effectiveness of cervical epidural injections.^1–5^

Foraminal or extraforaminal cervical disc herniation often leads to radicular pain over upper extremity by irritation or compression of dorsal root ganglion. Therefore, in patients with radicular pain, TF approach is preferred by physician to IL approach because it can deliver injectates directly around nerve root and dorsal root ganglion which is regarded as main pain sources.^6–8^

On the other hand, axial pain over neck or scapular area is originated from sinuvertebral nerve located in ventral epidural spaces, which has been described to be related to central or paramedian disc herniation. It is very questionable that TF injection is also more effective than IL injection in the patients with axial neck or interscapular pain. In lumbosacral disc herniation, TF injection has several advantages over IL injection in the patients with axial low back pain because TF approach enables the physician to advance the needle and deliver the medication directly to ventral epidural spaces from which axial back pain has been reported to be originated.^9^ But in cervical epidural injection, TF injection is performed in supine position and needle is advanced into posterior aspect of neural foramen in order to avoid vascular penetration and devastating side effects, therefore, even TF injection also has the limitation in direct administration of injectate into ventral epidural space.

To our knowledge, there is no literature comparing the clinical outcomes between TF and IL epidural injections in the patients with axial pain due to cervical disc herniation. The purpose of this study is to evaluate clinical efficacy of cervical epidural injection in terms of pain control and functional improvement in the patients with axial pain due to cervical disc herniation and to compare the clinical efficacy between TF and IL approaches.

## MATERIALS AND METHODS

### Patients

This study was approved by Institutional Review Board of our hospital. Patients who underwent cervical epidural injection from June 2013 to December 2013 for axial neck/interscapular pain which did not respond to oral medication and physical therapy at least for 4 weeks at Department of Physical Medicine and Rehabilitation were included in this study. Among them, the patients who met the following criteria were included: axial pain such as neck or interscapular pain without radiating pain over the upper extremities, central or paramedian cervical disc herniation at 1 or 2 segmental levels on magnetic resonance image (MRI), and no evidence of spinal cord compression or signal change on MRI. The MRI were evaluated and interpreted by radiologists who were expert in spinal problems. The patients with shoulder problems, neurological deficits, or other problems for cervical nerve root compression than cervical disc herniation including tumor or stenosis were excluded. Those with previous cervical epidural steroid injections within 3 months, previous cervical spine surgery were also excluded. We usually performed cervical medial branch block in patients with axial pain in advance to cervical epidural injections, in consideration of literatures reporting that facet joint pain was most prevalent cause of cervical axial pain.^10,11^ Thus, the patients who had shown significant pain reduction after medial branch block did not undergo epidural injection and not included in this study populations. Finally, 108 patients were included in this study. Among them, 56 underwent epidural injection by IL approach (IL group) and 52 underwent by TF approach (TF group). There were 19 male and 37 female patients in the IL group and 26 male and 26 female patients in the TF group. There was no significant difference regarding gender ratio between the IL and the TF groups.

### Interlaminar Epidural Injection

A patient was placed in a prone position on the fluoroscopy table with arms at the side. A blanket was placed under the chest. The neck was flexed with the head resting on a folded towel or blanket. Injection level was established in consideration of disc herniation levels. For example, injection was performed at the C5–6 level if disc herniation was found at C4–5 or C5–6 disc on MRI and at C6–7 level if lesion was placed at C6–7 disc on MRI. After skin preparation was done with povidone, the skin and subcutaneous tissues were anesthetized with 1 mL of 1% lidocaine at the entry point. A 20-guage Tuohy needle was inserted in the midline under fluoroscopic guidance. Loss of resistance was used to identify the epidural space. A syringe containing normal saline was attached to the Tuohy needle. Maintaining the same trajectory, the needle was advanced with checking loss of resistance under lateral C arm view. Subsequently, an epidurogram was performed using contrast material (Omnipaque 180, Amersham Health, Princeton, NJ) under the real-time fluoroscopy. Once it was determined that the dye spread in the epidural space and ensured that there was no intrathecal or intravascular pattern, the mixed injectate of 5 mg of dexamethasone (1cc) and 0.5% lidocaine (3cc) was slowly administered. After making sure that there was no problem for few minutes after injection of small volume (about 0.5cc) of mixture, the rest of mixture was slowly injected. After the needle was removed, the patient was observed for an appropriate length of time before being released from hospital.

### Transforaminal Epidural Injection

Transforaminal epidural injections were performed in the way that was introduced in another literature regarding cervical TF epidural injections written by ourselves.^12^ A patient was positioned supine on a table. His or her neck slightly was extended and the head was rotated away from the injected side. Treatment level was decided at lesion level detected on MRI and correspondent to clinical manifestation. The C-arm was rotated into a 45° to 60° ipsilaterally to injection side to produce the largest cross-sectional area of the foramen to be injected. After skin preparation, the skin and subcutaneous tissues were anesthetized with 1% lidocaine around needle insertion point. A 25-gauge spinal needle was advanced into the posterior–inferior aspect of neural foramen and just anterior aspect of superior articular process under the guidance of intermittent fluoroscopy. When the needle tip was contacted with anterior part of superior articular process adjacent to the posterior–inferior aspect of the foramen, the needle was advanced slightly under fluoroscopic guidance to locate the needle tip into the posterior–inferior foramen. At this point, we confirmed that the needle tip was not advanced beyond the mid portion of the pedicle in a true AP view in order to avoid needle penetration into the spinal canal. Contrast material (Omnipaque 180, Amersham Health) was slowly administered under the continuous fluoroscopy to confirm that the contrast media outlined the spinal nerve and spread along the medial border of the pedicle and the epidural space without intrathecal or intravascular pattern. After this, the medication of 5 mg of dexamethasone (1cc) and 0.5% lidocaine (1.5cc) was slowly injected. After ensuring that there was no problem for few minutes after injection of small volume (about 0.5cc) of mixture, the rest of mixed injectate was slowly infused. After the needle was removed, the patient was observed for an appropriate length of time before being released from hospital.

### Evaluation of Pain and Function

The evaluation of pain and function was conducted with the same way which was performed in another literature about clinical efficacy of cervical epidural injection written by ourselves.^12^ The pain intensity was evaluated by Numeric Rating Scale (NRS) ranging from 0 (no pain) to 10 (worst possible pain). All patients were asked to provide the average severity of their symptoms over a recent 1 week. The Korean version of Neck Disability Index (NDI, %) was used for assessing functional level.^13,14^ The Korean version of the NDI was validated as reliable measurement tool to evaluate the functional disability in Korean patients with cervical disorders.^15^ The patients were examined at pretreatment, 2 weeks, and 8 weeks to investigate pain reduction and functional improvement after treatment and to compare the difference of clinical outcomes between the IL group and the TF group.

Successful pain relief was defined if a 50% or more reduction of NRS score was achieved in comparison with pretreatment one. Successful functional improvement was defined if at least a 40% reduction of NDI was obtained.^16,17^

### Same Size Calculation

Sample size was calculated based on the literature comparing IL and TF in lumbar disc herniation. They showed that 70% of TF group had a meaningful improvement of NRS, while only 45% of IL group showed the same results.^9^ At least 30% difference in proportion of successful outcomes was established to be clinically meaningful in our study. Considering a 0.05 two-sided significance level, a power of 80%, and an allocation ratio of 1:1, and at least 42 patients in each group were required.

### Statistical Analysis

The statistical analysis was conducted with the same way which was performed in another literature about clinical efficacy of cervical epidural injection written by ourselves.^12^ Statistical analysis was performed using the SPSS Version 14.0 statistical package (SPSS, Inc., Chicago, IL). The number of disc herniations, gender proportion, and the proportion of successful NRS and NDI results after treatment were compared between the 2 groups using Chi-squared test. Comparison of age, duration of symptoms, and NRS and NDI at pretreatment between the 2 groups were conducted with Student *t* test. Results were thought to be statistically significant if the *P* value was <0.05.

## RESULTS

The IL group included 25 patients with 1 level and 31 with 2 levels of disc herniation and the TF group included 26 with 1 level and 26 with 2 levels of disc herniation, which had no statistically significant difference. The detailed distributions of disc herniation levels found in MRI were as follows: the IL group consisted of 5 at C4–5, 12 at C5–6, 8 at C6–7, 14 at C4–5 and C5–6, and 17 levels at C5–6 and C6–7. The TF group included 3 at C4–5, 15 at C5–6, 8 at C6–7, 12 at C4–5 and C5–6, and 14 levels at C5–6 and C6–7. As well, there was no statistically significance in terms of age, duration of symptoms, lesions distributions, and NRS and NDI (%) at pretreatment (Table 1).

**TABLE 1:**
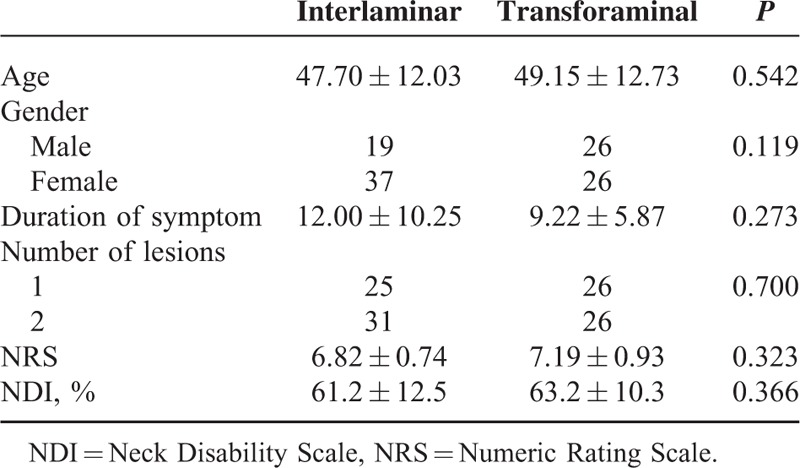
Comparison of Age, Gender Ratio, Duration of Symptom, Number of Lesions, NRS, and NDI (%) at Pretreatment

Overall, in terms of successful pain reduction measured by NRS, 79 (73.1%) among 108 patients showed successful results at 2 weeks, but those with successful results were reduced to 57 (52.8%) at 8 weeks after treatment. Similar results were found in successful functional improvement measured by NDI (%). At 2 weeks, 76 (70.4%) had successful results; however, only 52 (48.1%) showed successful functional improvement at 8 weeks after treatment.

**FIGURE 1:**
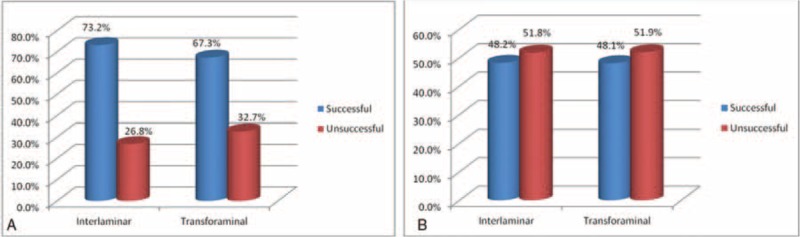
No significant difference was found between interlaminar and transforaminal approach in terms of Numeric Rating Scale at (A) 2 wk and (B) 8 wk.

**FIGURE 2:**
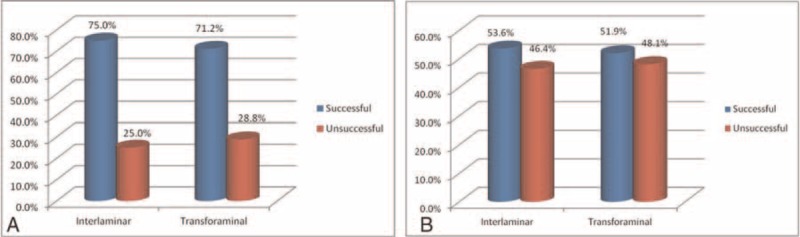
No significant difference was found between interlaminar and transforaminal approach in terms of Neck Disability Index (%) at (A) 2 wk and (B) 8 wk.

As to comparison between the IL and TF groups, no significant difference was found in proportion of successful results of NRS at 2 weeks (73.2% vs 67.3%) and 2 months (48.2% vs 48.1%) after treatment (Figure 1). Also, no significant difference was obtained in proportion of successful NDI between the 2 groups at 2 weeks (75.0% vs 71.2%) and 2 months (53.6% vs 51.9%; Figure 2).

Minor adverse events were observed in 5 (8.9%) and 6 (11.5%) patients with the IL and TF groups, respectively. Two patients had headache, 2 with dizziness, and 1 with increased pain in the IL group. Two patients had facial flushing, 3 with dizziness, and 1 with faintness in the TF group. All of them were transient and required no further treatment.

## DISCUSSION

Cervical epidural injection by IL and TF approaches has been reported to be an effective treatment in patients with symptomatic disc herniation or spinal stenosis.^1,2,18^ IL epidural injection obtained pain relief in 72.4% of patients with disc herniation or stenosis at 2 weeks after treatment.^2^ Approximately 60% of patients with cervical radiculopathy treated with TF approach obtained good clinical outcomes and consequently avoided surgery.^1,19,20^ A prospective study on clinical outcomes of TF approach for the treatment of patients with foraminal stenosis or disc herniation showed that 56% of patients obtained significant pain relief and resumed full activities at 6 months.^21^ Those studies showed variable outcomes because patient's characteristics, follow-up period, or number of treatment sessions were different each other. Although epidural injection was typically indicated in radicular pain due to stenosis or disc herniation, it was also performed in patients with cervical axial pain and showed clinical effectiveness.^4,22,23^

Our study demonstrated that both the IL and TF groups obtained successful clinical outcomes in over 70% of patients at 2 weeks and about 50% of patients at 8 weeks after treatment. Clinical benefits were reduced significantly at 8 weeks after treatment. It was stated that treatment effect deteriorated about at 2 or 3 months after epidural injection as steroid effects diminished.^24–27^ Especially, because dexamethasone used in present study has the trend toward shorter duration than methylprednisolone or triamcinolone, deterioration of treatment effects was expected to be so prominent at 8 weeks.^28^ This was why we established the relatively short follow-up period such as 8 weeks. But our purpose was not to investigate the duration of steroid effect, but to compare clinical benefits according to the different approaches before steroid effect deteriorated.

IL approaches were performed usually at C6–7 or C7-T1 level because dorsal epidural space was narrower at higher level and therefore, risk of intrathecal needle penetration was increased.^29–31^ We conducted IL approach at C5–6 in patients with C4–5 or C5–6 lesions with an attempt to deliver medication closer to lesion site as much as possible and no serious complications related to intrathecal penetration were not found.

TF approach was a preferred method to IL approach in treatment for radiating pain from cervical disc herniation because TF approach had the ability to place medication directly around the dorsal root ganglion involved in causing patient's radiating pain.^32^ On the contrary, in IL approach, injectate delivered in dorsal epidural space should spread into dorsal root ganglion to obtain clinical efficacy. But this was frequently blocked by foraminal disc herniation, which was 1 of the main pathologies in causing radiating pain.^32^ Expectedly, the study comparing clinical efficacy of TF and IL epidural injections in the patients with cervical radiculopathy due to disc herniation or cervical spondylosis revealed that 62% of TF patients and 16% of IL patients achieved pain relief.^33^

But in axial pain, which was mainly originated in ventral epidural spaces, it was questionable for TF approach to have more clinical efficacy than IL approach. Several reports had revealed that both IL and TF approaches obtained good clinical results in axial neck pain.^1,2,5,23^ But there has been no report to compare the clinical effectiveness between IL and TF injections in the patients with axial pain due to disc herniation. TF approach was performed in supine position and needle was approached toward posterior aspect of neural foramen in order to avoid vascular penetration of vertebral artery in cervical epidural injection. Therefore, TF approach had no advantage over IL approach because injectate usually could not be administered into ventral epidural space directly, which was a main different point from TF approach of lumbosacral epidural injection. In lumbosacral disc herniation, TF approach enables the physician to advance the needle and deliver the medication directly to ventral epidural spaces from which axial back pain has been reported to be originated.^9,34^ This property of cervical TF approach could explain that our study demonstrated no significant difference in clinical outcomes between IL and TF approaches.

The decision about treatment method was made on the basement of not only clinical benefits but also potential risks. Our study revealed that the IL and TF groups had minor adverse reactions in 8.9% and 11.5% of patients, respectively. The TF group had tendency to show slightly higher proportion of side effects, even if no significant difference was found between the 2 groups. Notably, TF approach could be associated with more serious side effects such as neurologic deficit because corticosteroid delivered by TF approach could be inadvertently injected into radiculomedullary artery and produced aggregated particles or embolus, which could lead to subsequent cerebellar, brainstem, or spinal cord infarct.^32,35^ Intra-arterial needle penetration could frequently occur even when needle was placed in appropriate position because vascular structures including radicular and vertebral artery were closely located at needle advancement route.^35,36^ Aside from particulated steroid-related complication, arterial dissection or vasospasm by direct needle trauma was another mechanism that could cause cerebellar or brainstem infarct in TF approach. As well, intravasation of local anesthetics could cause seizure or loss of consciousness.^36–39^ IL approach also produces serious side effects. Epidural hematoma was 1 of them, but it mainly occurred in patients who took anticoagulation therapy. One extensive review concluded that majority of adverse events of IL approach were minor and transient, serious complications such as spinal cord damage or intrathecal injection may also result but technique related, and therefore, IL was relatively safe procedures.^40^ It was assumed that in terms of serious complications such as death or brain infarct, TF was more relevant than IL.^41^

Our main result was that the TF approach had no advantage over the IL approach in clinical efficacy of the patients with axial pain from cervical disc herniation. Therefore, we suggested that the IL approach was more recommendable than TF approach which was more relevant to serious complications. Landa and Kim^41^ also stated in their review article that IL was more appropriate for the patients with axial neck pain, while TF was more appropriate for cervical nerve root irritation and IL injection might have lower complication rate than TF injections.

No serious side effects were observed in our study. This might be partly because dexamethasone, nonparticulate steroid, which was utilized in our study, rarely produced serious side effects or neurologic complications. Intravascular injections of particulate steroids, such as methylprednisolone and triamcinolone, had more potentials to produce the larger aggregates of particles, which could lead to vascular occlusion and devastating side effects.^42,43^

There were several limitations in this study. First, this study had no placebo group. Thus there could be criticism that it was difficult to differentiate natural pain remission from pain reduction by treatment effects. But we did not establish placebo group because main purpose of this study was to assess the difference of clinical efficacy according to the type of approach. Second, there could be opinion that 8 weeks follow up was not sufficient to evaluate the clinical implication of treatment method of epidural steroid injection. But we established 8 weeks follow-up period in this study because our purpose was not to investigate the duration of steroid effect, but to compare clinical benefits between 2 different approaches before steroid effect deteriorated.

Third, this study was retrospective design so that only the subjects who completed the 8 weeks follow up were included in this study. The possibility of selection bias about determination of injection route could arise. But injection route was chosen by randomly if patients’ symptom was mainly axial pain. As well, there was no significant difference in terms of general characteristics and level of disc herniations at pretreatment between 2 approaches. We assumed that there was no selection bias, which influenced our results.

## CONCLUSIONS

Cervical epidural injection showed favorable results at 2 weeks and moderate results in pain reduction and functional improvement at 8 weeks after treatment for the patients with axial pain due to cervical disc herniation. The IL and TF approaches showed no significant difference in clinical efficacy. Considering that TF approach was more related to devastating side effects than IL approach, it was suggested that IL approach was more recommended in axial pain originated from cervical disc herniation.
